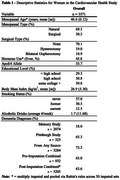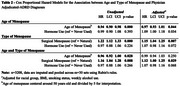# The Role of Reproductive Aging and Surgeries for Alzheimer's Disease and Related Dementias (ADRD) Diagnoses in the Cardiovascular Health Study

**DOI:** 10.1002/alz70860_105634

**Published:** 2025-12-23

**Authors:** Alexis Reeves, Oscar L Lopez, Michelle D. Shardell, Hoda S Abdel Magid, C. Elizabeth Shaaban, Michelle C Odden

**Affiliations:** ^1^ Stanford University, Stanford, CA, USA; ^2^ Cognitive Disorders & Comprehensive Alzheimer's Disease Center, Thomas Jefferson University, Philadelphia, PA, USA; ^3^ University of Pittsburgh, Pittsburgh, PA, USA; ^4^ University of Pittsburgh Alzheimer's Disease Research Center (ADRC), Pittsburgh, PA, USA; ^5^ University of Pittsburgh School of Medicine, Pittsburgh, PA, USA; ^6^ University of Maryland, Baltimore, MD, USA; ^7^ University of Southern California, Los Angeles, CA, USA; ^8^ Stanford University, Palo Alto, CA, USA

## Abstract

**Background:**

Most US individuals with ADRD are women. Menopause typically occurs in midlife, accompanied by declines in potentially neuroprotective and cardioprotective hormones. Earlier menopause leads to earlier exposure to decreased sex hormones, potentially increasing ADRD risk. Surgical menopause via reproductive surgeries may cause a sudden loss of hormones and may represent be a modifiable risk factor for ADRD. We aimed to determine if menopausal type and age are associated with physician adjudicated‐ADRD diagnoses.

**Methods:**

Data were from 3371 women prospectively followed from age 65+ years. Participants self‐reported age of menopause and/or surgeries, hormone‐therapy, education, and race (Black/White). For this study, given the bleeding criteria for determining final menstrual period, both hysterectomy and/or bilateral oophorectomy were considered surgical menopause. Adjudication for ADRD was conducted in two sub‐samples (*n* = 2397) by neurologist/psychiatrist committee review using neuropsychological tests, neurological examinations, medical records, physician questionnaires, and proxy/informant interviews. All participants had auxiliary information including medication, ICD‐9 codes, proxy reports, and death certificates. For women not included in the physician‐adjudicated sub‐sample, multiple imputation was used to impute adjudicated ADRD status and age of diagnoses (bounded regression) using auxiliary information. Cox proportional hazard models were used for estimations controlling for 1) hormone use and 2) additionally demographics and health behaviors.

**Result:**

The mean menopausal age was 46.6 years (SE=0.12); 30.5% of participants underwent surgical menopause (19.0% via hysterectomy and 10.9% via bilateral oophorectomy), and 45% used hormone therapy. After imputation, 63.6% were diagnosed with ADRD (Table 1). Older menopausal age was associated with a lower hazard of ADRD controlling for hormone use (HR=0.94[0.90,0.98]) and fully adjusted (HR=0.97[0.93,1.01]). Surgical menopause was linked with 1.22 [1.12,1.33] higher hazard of ADRD controlling for hormones, and 1.15 [1.04,1.25] higher hazard with full adjustment. In an additional model with age and type of menopause included together, menopausal age was non‐significant and surgical menopause remained positively associated with ADRD (HR=1.12 [1.00,1.25]).

**Conclusion:**

Although further work is needed to obtain more precise measures of menopause, results suggest older menopausal age may modestly reduce ADRD risk non‐independent of type, and that reproductive surgeries are associated with a higher risk of ADRD.